# Geographic disparities of cardiovascular and cancer mortality in the USA: 1981–2019

**DOI:** 10.1093/pubmed/fdad089

**Published:** 2023-07-01

**Authors:** V Lebakula, A G Cosby

**Affiliations:** Geospatial Science and Human Security Division, Oak Ridge National Laboratory, 37831-6017 Oak Ridge, TN USA; Social Science Research Center, Mississippi State University, 39762, Starkville, MS, USA

**Keywords:** Geographic mortality disparities, Cancer mortality, Cardiovascular mortality, Leading causes of death, Mortality among US counties

## Abstract

**Background:**

Cardiovascular and cancer mortality are the two leading causes of death in the developed world including the USA. However, mortality trends for these diseases are highly dynamic, and the geographic landscape is in transition. We analyze patterns of mortality improvement at county level during recent decades focusing on mortality decline and geographic diversity.

**Methods:**

We grouped age-adjusted mortality rates of cardiovascular and cancer diseases from CDC WONDER for 2959 US counties into 3-year time periods to improve reliability. We calculated percent mortality decrease between 1981–83 and 2016–19 for both causes to quantify mortality improvements for counties.

**Results:**

Using standard deviation as an index of disparities, place-based cancer mortalities were 68% larger than cardiovascular disparities. Significantly, 566 US counties had same or higher rates of cancer mortality in 2019 as in 1981. The geographic distribution of mortality improvement in either cause tends to favor largely populated areas along coasts. Less-populated, rural places in the interior and southeastern regions were experiencing less improvement.

**Conclusions:**

At the county level, large place-based disparities exist for both causes of death with the magnitude of disparities being substantially larger for the reduction in cancer deaths. Put differently, place matters, more for cancer than cardiovascular mortality.

## Introduction

For decades, cardiovascular disease has been the leading cause of death in the USA followed by cancer. As recently as 2019, these two diseases accounted for nearly 45% of the nation’s deaths.^1^ Fortunately, these causes of death have been declining significantly; between 1981–83 and 2017–19, cardiovascular mortality declined by 57.33% and cancer by 27.43%. However, using only national trends masks profound differences in mortality patterns at the local level.^2–7^ An analysis of patterns and trajectory of mortality improvement at a more spatially specific level can provide context for understanding mortality change and may provide the basis for projecting future mortality. To that end, we contrast the rates of change and patterns of spatial disparities for cardiovascular and cancer mortality for 2959 places (US counties) between 1981–83 and 2017–19.

## Methods

Cardiovascular and cancer mortality data were obtained from the Centers for Disease Control and Prevention (CDC) National Center for Health Statistics (NCHS) (https://www.cdc.gov/nchs/index.htm). We combined two databases available in the CDC WONDER: the Compressed Mortality File (1981–2016) and the Underlying Cause of Death File (2017–2019).^8^ The Compressed Mortality File and Underlying Cause of Death File are considered representative of the population of US mortality for each year, and the coverage rate approximates 100% of US mortality. The data are based on the US Vital Statistics Cooperative Program where the National Center for Health Statistics codes original death certificates provided by state registration offices. Deaths are coded according to the International Classification of Diseases codes. We consider the two files to be sufficiently homogenous to merge since both approximate the US mortality for specific years; both are based on death certificates and coded with ICD codes. The combined datasets were based on over 35 million cardiovascular deaths and over 21 million cancer deaths that occurred in the USA between 1981 and 2019.^8^ From these datasets, we selected mortality data (ICD-9 and ICD-10) codes for diseases of the circulatory system and neoplasms. Cancer mortality includes deaths from all neoplasms categorized in ICD-10 coding into 98 malignant, 10 in situ, 27 benign and 12 unknown or uncertain cancer types. Cardiovascular mortality includes deaths from all the circulatory diseases categorized in 1CD-10 coding into acute rheumatic, chronic rheumatic heart diseases, hypertensive diseases, ischemic heart diseases, pulmonary heart disease, other forms of heart disease, cerebrovascular diseases, diseases of arteries, arterioles and capillaries, diseases of veins, lymphatic vessels and lymph nodes, not elsewhere classified and other and unspecified disorders of the circulatory system. The population consisted of compressed mortality data at the county level (*n* = 2959) that reported both cardiovascular and cancer rates. To improve reliability for smaller populations, we grouped mortality data into 3-year periods. Cardiovascular and cancer rates for the 3-year periods were calculated per 100 000 and adjusted to the year 2000 standard million to account for age structure differences via CDC WONDER analytics. NCHS grouped deaths into 11 age groups to calculate the age-adjusted rates using the direct method. Methods to calculate age-adjusted rates (refer to the equation below) are documented on CDC Wonder website.^9^^,^^10^


$$ Age\ adjusted\ mortality\ rate={\sum}_{g=1}^{11}\frac{SP_g}{SP}\ast{R}_g $$

where ${SP}_g$ is the standard population for each age group *g* (*g* varies from 1 to 11); $SP$ is the total standard population of the USA and ${R}_g$ is the age-specific death rate for age group *g*.

Changes in cardiovascular and cancer mortality were calculated as percent change in age-adjusted mortality rates between 1981–83 and 2017–19. Mortality improvement or decline is defined as positive percent decrease.


$$ \Delta M=\frac{M_{\left(1981-1983\right)}-{M}_{\left(2017-2019\right)}}{M_{\left(1981-1983\right)}}\ast 100 $$

where *M* is the age-adjusted mortality rate per 100 000; $\Delta M$ is the percent change in age-adjusted mortality rates per 100 000 between the 1981–83 and 2017–19 periods and $\Delta M$ is calculated for both cardiovascular and cancer mortality percent change for each of 2959 counties.

We constructed a series of GIS maps using ESRI ArcView software to visually depict the geographic spread of mortality trends for US counties over the timeframe of the study. We used the national trend rates of mortality decline for cardiovascular improvements (57.33%) and cancer improvements (27.43%) as standards of comparison for each county. Our maps depict (i) counties with improvements in both cancer and cardiovascular mortality exceeded the national estimate, (ii) counties with improvements for both causes are less than the national estimate, (iii) counties in which cardiovascular improvement exceeded the national average and (iv) counties in which cancer improvement exceeded the national level.

## Results

**Fig. 1:**
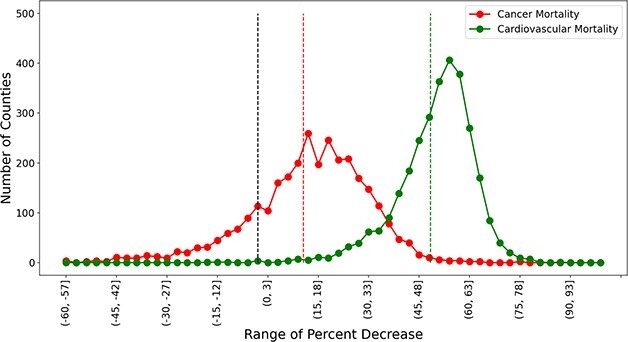
Grouped frequency distribution plot of percent decrease in cardiovascular and cancer mortality of 2959 counties in the USA: 1981–2019. The cancer improvement curve is in red, and the cardiovascular improvement curve is in green. Red and green dotted lines represent the county mean percent decrease in cancer and cardiovascular mortality, respectively. The black dotted line is a zero line and decrease in mortality rates was observed only on the right-hand side of this line.

First, US counties had a much higher improvement in cardiovascular mortality than cancer mortality (Fig. 1) for the period 1981–83 to 2017–19. Mean improvement rates for US counties during this period were 51.55% for cardiovascular and 13.37% for cancer mortality. The majority of the US counties (2938) had larger cardiovascular improvement rates than mean cancer rates. Only 19 counties had cancer improvements exceeding mean cardiovascular rates. Please note that the national mortality rates (reported earlier) of 57.33% for cardiovascular and 27.43% for cancer substantially exceed the mean county rates. The reason for these differences is that county-level mortality means do not account for differences in county population, whereas national means are based on the total US population.

Second, we interpret the standard deviation (SD) of each distribution as an index of spatial disparities among counties (Fig. 1). While an examination of cardiovascular and cancer improvement rates reveals considerable place-based variations, the spatial disparities between counties were more pronounced for cancer. The SD for the cancer improvement distribution equaled 18.73, and the SD for cardiovascular improvement distribution equaled 11.14, indicating 68% more spatial disparity for cancer improvement.

Third, it is important to note that some counties did not experience improved rates for either cause (Fig. 1). No improvement in cancer mortality was found in 566 counties; only 7 counties had no improvement in cardiovascular mortality. Improvement in both mortalities was seen in 2390 counties. A total of 562 counties exhibit improvement in cardiovascular mortality but not cancer, whereas there were only three counties with improvement in cancer alone.

**Fig. 2:**
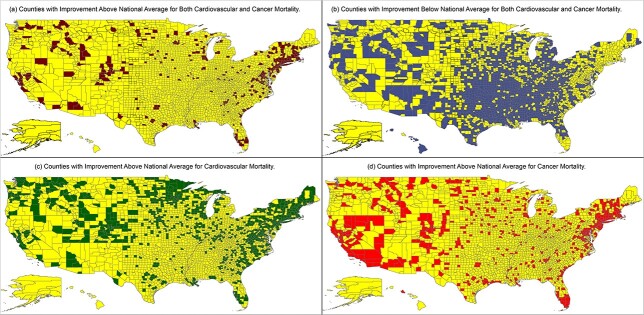
Geographic disparities in age-adjusted mortality improvement in cardiovascular and cancer diseases in the USA: 1981–83 through 2017–19. Footnote: These maps portray county-level disparities in the rate of cardiovascular and cancer mortality for US counties for the period 1981–83 through 2017–19. In each map, county rates of change are compared with national rates of change where national improvement for cardiovascular was 57.33% and national improvement for cancer was 27.43%. (**a**) The maroon-shaded counties exceeded the national average in both cardiovascular and cancer mortality. (**b**) Blue shading indicates counties below the national average for cardiovascular and cancer. (**c**) Green shading indicates counties with cardiovascular improvements exceeding the national average, and in (**d**) red shading indicates counties with cancer exceeding the national average.

Fourth, in Fig. 2a, we have depicted counties whose improvement rate for both cardiovascular and cancer mortality exceeds the comparable national rates. Of the 2959 counties with sufficient mortality data, only 350, or 11.83%, exceeded the national rate for both diseases. Among several general patterns that can be noted is the concentration of high improvement locations along the heavily populated Northeastern Corridor generally spanning from Boston through New York City to Washington DC. Similar coastal concentrations are seen in the populated San Francisco, Las Angeles and Miami regions. In Fig. 2b, we depict counties where cardiovascular and cancer mortality rates were less than the national rate of improvement. There were 1752, or 59.21%, US counties below the national rate. While low-improving counties were found across the USA, the heaviest concentration tended to be in the central and southeastern regions, which included numerous less-populated counties.

Fifth, in Fig. 2c, we provide map that illustrates the patterns of cardiovascular mortality rates that exceed the national rate of improvement. There were 937 counties or 31.67% whose cardiovascular rates were improving. The remaining 2022 or 68.33% of counties were experiencing lower or equal rates in comparison with the national figures. In a similar fashion, Fig. 2d illustrates the geographic spread of counties that exceeded the national rate of improvement for cancer mortality. There were 620 counties, or 20.95%, where cancer mortality improvements exceeded the national estimate and 2339 that had lower or equal rates of improvement. The geographic distribution of improvement in either cardiovascular or cancer mortality tends to favor largely populated areas along the Atlantic and Pacific coasts. Less-populated, rural and nonmetropolitan places in the interior and southeastern regions were often experiencing less improvement.

## Discussion

### Main findings

Place-based variations, in this case at the county level, in cardiovascular and cancer mortality trends are important components in understanding the complexity of the USA overall improvement. National-level mortality declines were achieved or exceeded in relatively few counties (31.67% for cardiovascular and 20.95% for cancer). The county-level disparities were substantial with cancer trend variations being 68% larger than the spatial disparities for cardiovascular mortality. Mapping the county-level trends revealed that improvements tended to be concentrated in a minority of counties that were often located in the largely populated areas of the Atlantic and Pacific coasts and that low or no improvement areas tended to be in the interior and southeastern regions. Geography in the USA seems to have a great deal to do with your chances of dying from cardiovascular or cancer diseases.

### What is already known on this topic?

There is a massive amount of scientific literature that focuses on cardiovascular and cancer diseases that is clearly beyond the scope of our short report.^6^^,^^7^ It is broadly understood that there has been a long-term improvement in cardiovascular health that has resulted in significant declines in the rate of cardiovascular mortality in the USA. Cancer mortality trends have been more complex. Cancer mortality has been increasing for most of the twentieth century and reached its peak for the nation in the early 1990s, and then began to consistently decline. Our study builds on this knowledge by investigating the disparities in trends at a lower level of geography.

### What this study adds

Our study quantifies the considerable place-based disparities in improvement rates for both cardiovascular and cancer mortality including the observation that county-based disparities in improvement were much greater for cancer than cardiovascular diseases. Mapping of these data also identifies the fewer places in the USA that were meeting or exceeding the national rates of improvement. These findings taken together argue for the need to take into account geographic rates of improvement in assessing health progress and, possibly, as a lens for help in understanding health disparities.

### Limitations

As with many ecological health studies, we were unable to distinguish or measure individual, family and neighborhood effects. For example, are declines in mortality concentrated among certain individuals, families or neighborhoods? This is certainly a limitation in counties with very large and diverse populations. Also, there is concern with underestimating the level of disparities in counties with very small populations. There are also numerous explanatory and adjustable confounders that could lead to a better understanding of the spatial disparities among counties. These include measures of social determinants of health (e.g. wealth and education), race and ethnicity, quality and accessibility of healthcare, environmental influences (e.g. exposure to carcinogens and water/air quality) and behavioral risk factors (e.g. tobacco use, diet and exercise).

Place in the USA has profound consequences for improvement in both cardiovascular and cancer mortality. On average, US counties were experiencing higher decline and fewer disparities in cardiovascular mortality. While there has been progress in reducing mortality of both diseases, cardiovascular mortality was declining more rapidly with fewer place-based disparities. Although the social, cultural, biological, genetic and environmental characteristics were constant within each county, we found great differences in the improvement rates and disparities between these causes. Why should some places benefit more from the nation’s trend in reducing cardiovascular mortality, and why should benefits be more disparate for cancer?

It can be noted that greater improvement in cardiovascular mortality is linked to fewer place-based disparities. Arguably, knowledge, technology and adoption exist in the higher-performing counties that could transform cardiovascular and cancer outcomes if effectively diffused among lower-performing counties. From a data science perspective, we can improve outcomes by reshaping the improvement curve via a process of diffusion and adoption.

## Conclusions

In recent decades, there has been substantial progress in improving the nation’s cardiovascular and cancer mortality rates. As a nation, USA had learned a great deal about public health policy, behavioral risk factors, screening, medical intervention and environmental risk factors that can result in reduced mortality from these diseases.^11^^,^^12^ An examination of patterns of improvement at the local level is more troubling. Benefitting from national trends was strongly associated with where you live. Since the USA can dramatically reduce national cardiovascular and cancer mortality, why are places not taking advantage of this knowledge? There are certainly opportunities for adoption and diffusion using this existing knowledge. The core conclusion of this study is that there are profound geographic differences in mortality improvement for two major causes of death in the USA and that only a few counties have improvements that equal or exceed the national trends. The greatest declines in cardiovascular and cancer mortality have a strong geographic component; the benefits are concentrated in the heavily populated urban centers along the US coasts.

Place made a great difference in cardiovascular and cancer mortality. There were 566 counties in the USA that had the same or higher death rates from cancer in 2019 than in 1981, whereas only seven counties had the same or higher death rates for cardiovascular mortality. These 566 high-cancer-risk counties should be a priority for research that focuses on place-based factors and, perhaps, would serve as a laboratory for understanding the challenges of adoption and diffusion approaches.

This possibility forms a backdrop for evaluating future health priorities and policies. This short report is an argument for including place-based variations in health benefits as an important component in developing public health strategies.

## Notes

**V. Lebakula,** Research Scientist

**A.G. Cosby,** William L. Giles Distinguished Professor Emeritus
